# 

**Published:** 1874-12

**Authors:** 


### CONTENTS:

### ORIGINAL COMMUNICATIONS.

ARTICLE I.—Epithelial Cancer of the Tongue. By Dr. Hubbard .......... 337

ARTICLE II.—Dr. C. S. Smith on Arthur's Treatment. .......... 344

ARTICLE III.—East Tennessee Dental Association .......... 345

### SELECTED ARTICLES.

ARTICLE IV.—American Dental Association. (Continued.) .......... 347
ARTICLE V.—On the Condition of the Mouth and Teeth During Pregnancy. By Oakley Coles .......... 361
ARTICLE VI.—Cicatricial Contraction from Burns, Involving the Face. By Gurdon Buck, M. D .......... 369

### EDITORIAL, ETC.

Virginia State Dental Association .......... 375
Celluloid .......... 376
The Cause of so Many Failures .......... 376

### OBITUARY.

Dr. George E. Hawes .......... 377

### MONTHLY SUMMARY.

Local Use of Chloral .......... 378
Cutting Teeth .......... 378
Lead as a Constituent of Enamel the Cause of Chronic Poisoning .......... 380
How to Insert the Hypodermic Syringe .......... 380
Arsenic Poisoning by a Green Carpet .......... 381
How to Deprive Iodine of its Stain .......... 381
The Prevention of Dental Caries .......... 381
Monobromide of Camphor .......... 381
To Suppress Hemorrhage of the Mouth During Operations .......... 382
Electricity in Vomiting .......... 382
Death from Chloroform .......... 382
Physical Peculiarities of Negroes .......... 382
To Arrest Hemorrhage of Mouth and Gums .......... 383
Nothing New Under the Sun .......... 383
Lancing the Gums .......... 383
Directions for Escaping Colds .......... 384

